# Reference Intervals in Combined Veterinary Clinical Examinations of Male Black-Spotted Pond Frogs (*Pelophylax nigromaculatus*)

**DOI:** 10.3390/ani11051407

**Published:** 2021-05-14

**Authors:** Jun-Kyu Park, Jeong-Bae Kim, Yuno Do

**Affiliations:** 1 Department of Biological Science, Kongju National University, Gongju 32588, Korea; pjk8578@smail.kongju.ac.kr; 2 Marine Environment Research Division, National Institute of Fisheries Science, Busan 46083, Korea; jbkim347@korea.kr

**Keywords:** bacterial killing assay, blood serum chemistry, radiographic technique, veterinary clinical examination

## Abstract

**Simple Summary:**

The reference intervals (RIs) of immunity, serum components, bone mineral density (BMD), and body composition in 151 males of *Pelophylax nigromaculatus* were established. These analyses are easily replicable and can safely and accurately diagnose the physiological condition of animals. The use of combined examination allows for the establishment of a successful conservation strategy through the identification of conservation problems in many vertebrate groups.

**Abstract:**

In conservation physiology, analyzing the physiological response of an organism to understand its ability to adapt to environmental changes is a key technique in establishing a successful conservation strategy. Veterinary clinical examinations determine the physiological condition of animals accurately and safely, and this examination is synergistic when combined. The accuracy and safety of a clinical examination makes it advantageous for use in amphibians with high species diversity and numerous endangered species. However, it is necessary to establish a reference interval (RI) for precise interpretations and identification of animals with abnormalities through individual unit testing. We have established RIs for the immunity, serum components, bone mineral density (BMD), and body composition of black-spotted pond frogs (*Pelophylax nigromaculatus*). Black-spotted pond frogs are a common species and are widely distributed in East Asia, with suitable characteristics for environmental monitoring. Serum was extracted from 151 male frogs to establish the RI for bacterial killing ability in order to represent immunity. We also used the serum to establish an RI of ten additional serum components to determine the nutritional status, organ function status, body osmotic pressure, and homeostasis conditions. The BMD and three body composition measures for diagnosing food intake status and nutritional condition were measured using dual energy X-ray absorptiometry. The RI was recorded as the mean ± standard deviation, median, first (25%) to third (75%) quantile range, 95% confidence interval of the mean and median, and the 95% percentile (2.5%–97.5% range) of all components. The use of combined clinical veterinary examinations aids our understanding of the physiological conditions of an individual according to biotic and abiotic factors on a complex spatiotemporal scale in an ecosystem.

## 1. Introduction

In conservation physiology, understanding the physiological conditions and responses of animals to environmental changes allows for the rapid assessment of conservation problems to establish a successful conservation strategy [1]. Veterinary clinical examinations, including veterinary laboratory medicine (clinical biochemistry, hematology, and immune assay) and veterinary diagnostic radiology (dual-energy X-ray absorptiometry and micro-CT) can be used to diagnose the physiological condition of animals in changing environments [2,3,4,5,6,7]. These methods can be safely analyzed without fatalities, and provide an accurate diagnosis of the animal’s condition. For example, bacterial killing assays using plasma or serum indicate innate immunity levels through complement reactions, which have been used to test the immune response in various animals, such as bats, birds, and amphibians, via acute stress, physiological stress, or dietary proteins [8,9,10,11,12]. Blood chemistry analysis analyzes the hepatic or renal function, homeostasis conditions, osmotic pressure in the body, and nutritional status of many animals against the backdrop of environmental changes such as salinity, temperature, or oxidative stress [13,14,15,16]. In addition, radiographic techniques have been used on various animals with disease, captive stress, or predator interaction, to diagnose physical abnormalities, nutritional deficiencies, bone fractures, and food intake status [17,18,19,20]. However, these examination components vary in terms of their sensitivity level and response factors with the type of environmental factor or the stress intensity. Combining various veterinary clinical examinations can aid in diagnosing the physiological condition of animals in more detail.

Among the higher vertebrates, many amphibians are seriously endangered and suffer from population decline [21]. The main declining factors are environmental variables (large-scale pandemics, climate change, pollutants, habitat destruction, and the introduction of invasive species) that can promote rapid changes in amphibian physiological conditions [21,22]. Therefore, physiological conservation research on amphibians is required [22]. However, a standard diagnostic procedure for health and physiological conditions does not exist for amphibians. These problems can be overcome through the application of a veterinary clinical examination that can safely and accurately diagnose the health and physiological condition of animals. For many amphibians undergoing a population decline, the safety of examination makes the analysis more appropriate [23,24]. Previously, a single veterinary clinical examination to identify physiological responses to environmental stress in amphibians has been applied [15,25,26,27]. These studies were conducted for the purpose of comparison analyses among groups. In addition to safety and accuracy, veterinary clinical examinations have the advantage of being able to diagnose an individual or group without the comparison group by setting the reference intervals (RI). RIs are needed to support the interpretation of quantitative pathology results [28], and aid in identifying each outlier individual, which are those individuals that fall outside of the normal health range, by diagnosing and manipulating the physiological conditions [4,29]. The advantage of veterinary clinical examinations that diagnose via individual units is maximized in groups with many endangered species, where it is difficult to obtain a large number of individuals. Therefore, the RI can be particularly important in amphibians that have high species diversity and a large number of endangered species, which should be established in these groups. The lack of RI—due to the limited number of studies and the unfamiliarity of amphibians for researchers and veterinarians—make amphibian analyses and comparisons more difficult [23,24]. To solve this problem, studies to establish an RI for veterinary clinical examination must be conducted using various amphibian species.

In this study, we aimed to establish an RI for immunity, body composition, bone mineral density (BMD), and serum chemistry components for combined veterinary clinical examinations in black-spotted pond frogs (*Pelophylax nigromaculatus*). This semi-aquatic frog is widely distributed in East Asia and is a common species in most freshwater ecosystems. The frogs are very easy to collect in both breeding and non-breeding seasons as they are not rare and are philopatric to breeding sites [30]. Owing to the ease of breeding and raising this species in the laboratory, they can be easily used for identifying the physiological and genetic patterns in frogs caused by environmental change [31]. Hence, these frogs can be employed to aid our understanding of changes in frog health with the environment of the habitat, and for monitoring or experimenting with long-term and short-term environmental changes. Moreover, their body composition and abnormalities can be detected without using X-rays. This species has sufficient blood volume for analysis due to the large body size of adult frogs (59.3–89.4 mm), which is important for veterinary clinical examinations. Therefore, we used the 95% confidence interval (CI) and 95% percentile range to confirm the RI of the components determined, using veterinary clinical laboratory medicine and diagnostic radiography to assess black-spotted pond frogs.

## 2. Materials and Methods

### 2.1. Animals

A total of 210 black-spotted pond frogs (*Pelophylax nigromaculatus*) were collected from a field in Chungcheongnam-do between May 2020 and July 2020. These frogs were hand captured between 7 p.m. to 11 p.m. from 68 sampling sites (1–3 individuals per sampling site). We collected frogs from paddy fields in various environments (altitude, habitat structure, natural landscape, and land use) to avoid restricting the data to the environmental characteristics of a specific location. All frogs were captured during the breeding season; only male frogs with complete secondary sexual differentiation were collected. The adult males were identified by the presence of a vocal sac and nuptial pad on the first finger, which are secondary sexual characteristics of this species. We measured the snout–vent length (SVL) and body weight as the basic physical information of the individual. The SVL was measured as 0.01 mm units using a digital caliper (IP54, SHAHE Digital Caliper, Wenzhou, Zhejiang, China), and the body weight was measured as 0.01 g units using a digital balance. Animals were euthanized by pithing after analysis and stored in 70% ethanol. Experimental procedures on animals were conducted in accordance with the regulations and approval of the Experimental Animal Ethics Committee of Kongju National University (KNU_2019-01).

The RIs were established by following the guidelines of the American Society of Veterinary Clinical Pathology (AVSCP) [32]. Individuals with an abnormal appearance were identified and removed from the analysis. We detected outliers using the 1.5 interquartile range. If one component value among all veterinary clinical examination items was detected as an outlier, it was excluded from the analysis. As a result, the component values from 59 individuals (of the 210 individuals collected in total) were removed and, subsequently, the values of 151 frogs were used for the establishment of RIs in veterinary clinical examinations.

### 2.2. Immune Assay

As chemical anesthesia physiologically affects the muscle tissues of frogs [33], and supplementing anesthesia with ice-cold water helps to stop ectothermic vertebrates, such as amphibians and reptiles, from experiencing pain [34], for the immune assay, frogs were anesthetized in ice-cold water and blood was extracted by cardiac venipuncture. The extracted blood was transferred to a serum separator tube (SST) and centrifuged at 3000× *g* for 10 min. The supernatant was extracted and stored at −40 °C until analysis.

The bacterial killing assay was performed according to the methods described previously [35]. Serum samples were diluted 1:20 in Ringer’s solution (10 µL serum: 190 µL Ringer), and mixed with 10 µL of non-pathogenic *Escherichia coli* (Microbio-Logics #24311-ATCC 8739, Minnesota, MN, USA) working solution (approximately 10^4^ microorganisms). The positive control involved mixing 10 µL of *E. coli* working solution in 200 µL of Ringer’s solution. The negative control contained only 210 µL of Ringer’s solution. All samples and controls were incubated for 60 min at 37 °C. After incubation, 500 µL of tryptic soy broth (TSB) was added to the samples and controls. We mixed the bacterial suspensions and transferred the solutions to 96-well microplates with 300 µL duplicates. The transferred solutions were incubated for 2 h at 37 °C. The bacterial optical densities were measured hourly using a microplate spectrophotometer (wavelength 600 nm) for a total of four readings. The BKA was calculated at the beginning of the bacterial exponential growth phase according to the formula: [(1 − (optical density of sample/optical density of positive control)], which indicates the proportion of killed bacteria in the serum samples compared to the positive control.

### 2.3. Measurements of Body Composition and Bone Mineral Density

**Figure 1:**
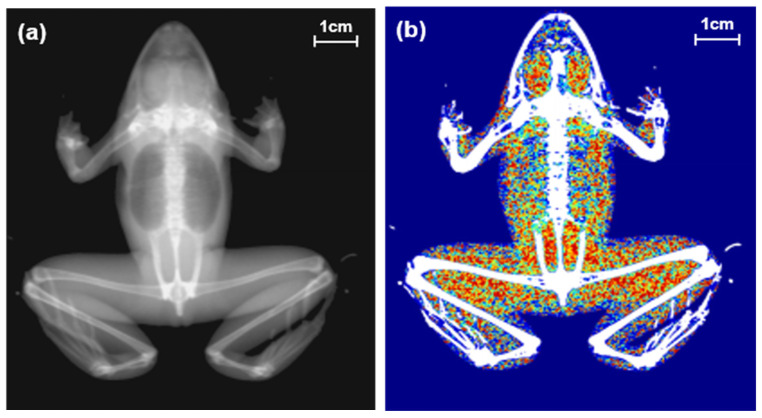
Two types of images of a black-spotted pond frog using dual energy X-ray absorptiometry: (**a**) body image; (**b**) composition image (green areas: lean body contents, red areas: fat contents).

Dual energy X-ray absorptiometry (DEXA; Medikors, InAlyzer, Seongnam, Korea) was used to measure body composition and bone mineral density (BMD). The bone mineral content (BMC), fat content, and lean body content was calculated by alternating high- and low-energy X-rays (Figure 1). The lean body content was obtained by subtracting the fat content from the tissue content (the sum of body water and muscle area). Frog samples were processed for at least three months in 99.5% methanol to minimize changes in body water content after blood extraction. We acquired three types of body composition data (BMC, fat content, and lean body content), mass (g) and ratio (%) using DEXA.

### 2.4. Serum Chemistry Analysis

After frogs were anesthetized in ice-cold water, within two hours of acclimatization, blood samples were extracted from frogs (equivalent to less than 1% of the frogs’ body weight) by cardiac venipuncture. Samples were transferred to a serum-separating tube (SST) and the blood was centrifuged (3000× *g*, 10 min). The supernatant was extracted to obtain the serum samples, which were later stored in a freezer at −40 °C until chemical analysis. A clinical chemistry automated analyzer (Hitachi Automatic Analyzer 7020, Japan) was used to analyze ten serum components: glucose, aspartate aminotransferase (AST), alanine aminotransferase (ALT), blood urea nitrogen (BUN), creatinine, total protein (TP), albumin, total globulin (TGB), calcium, and phosphate. TP, albumin, and TGB were used to confirm the nutritional status, protein metabolism, homeostasis, and status of renal and hepatic functions.

Glucose is used to determine the nutritional status of frogs [36], their metabolic state, stress [16], and the osmotic pressure in their body fluids [37]. ALT is a liver-specific indicator, whereas AST is a non-specific indicator of liver function [16]. Based on changes in ALT levels in isolation, it is difficult to determine the condition of the liver function, as this enzyme is extremely sensitive. AST is less sensitive; however, it is a non-specific indicator that varies with damage to the liver, heart, and skeletal muscle. Therefore, when ALT and AST are used in combination, they indicate stress in the hepatic functions [16]. BUN is produced from the waste product of protein metabolism [38]. Creatinine is produced from creatine, which is an energy source used during the muscle metabolism of glucose [39,40]. BUN and creatinine represent protein metabolism and muscle metabolism, respectively and, when used simultaneously, indicate the function of the kidneys [38]. The concentrations of TP, albumin, and TGB represent homeostasis status, malnutrition, blood loss, and changes in liver and renal function [16]. Calcium and phosphorus are used for identifying nutritional status and renal disease [41].

### 2.5. Statistical Analysis

In a veterinary clinical examination, the 95% confidence interval (CI) of the mean or median is generally used for components with a normal distribution. However, the CIs of the mean and median are different in components with a non-normal distribution. In this case, we use the logistic or square root transformation, or employ a percentile that is not significantly affected by outliers [29]. Therefore, in addition to the 95% CI of the mean and median, the 95% percentile range is described in components with non-normal distributions. Normality tests were performed using the Anderson–Darling normality test. We calculated the mean ±standard deviation (SD), median, first (25%) to third (75%) quantile range, 95% CI of the mean and median, and 95% percentile (2.5–97.5% range) of SVL, weight, immunity, body composition, BMD, and ten serum components in black-spotted pond frogs. The box-and-whisker plots were used to visualize the distribution of SVL and body weights. All analyses and calculations in the RI were performed using GraphPad Prism (GraphPad Software, version 8.00, San Diego, CA, USA). 

## 3. Results

### 3.1. The Basic Physical Information from 151 Male Frogs

**Figure 2:**
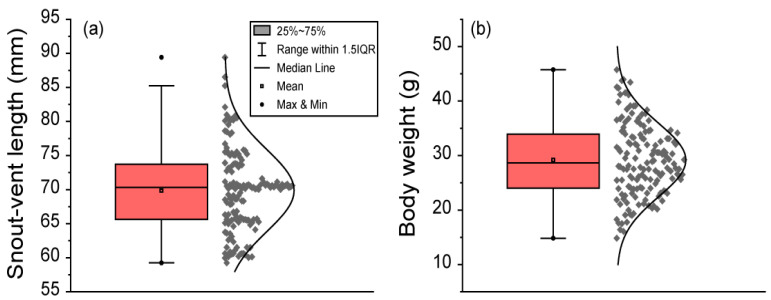
The body length and body weight of 151 male black-spotted pond frogs: (**a**) the snout–vent length (SVL) in frogs; (**b**) frog body weight. Box plots show the mean (central square point), median (central band), minimum (bottom dot), maximum (top dot), 25th and 75th percentiles (bottom and top of boxes), and range within the 1.5 interquartile range (IQR).

The SVL of frogs ranged from 59.25 to 89.42 mm. The mean ± SD of SVL was 69.87 ± 6.01 mm. The median SVL was 70.32 mm. The percentile range between 25% and 75% of SVL ranged from 65.62 to 73.72 mm. The 95% CI of mean SVL ranged from 68.90 to 70.83 mm. The 95% CI of the median SVL ranged from 69.10 to 70.61 mm. The 95% percentile of SVL ranged from 60.48 to 80.57 mm. Body weight ranged from 14.83 to 45.76 g. The mean ± SD of body weight was 29.19 ± 6.74 g. The median body weight was 28.67 g. The percentile range between 25% and 75% of body weight ranged from 24.02 to 33.94 g. The 95% CI of the mean body weight ranged from 28.10 to 30.27 g. The 95% CI of the median body weight ranged from 27.45 to 30.52 g. The 95% percentile of body weight ranged from 18.73 to 41.51 g (Figure 2).

### 3.2. Reference Interval of Immunity, Body Composition, and BMD

The reference intervals of immunity (BKA), body composition (mass and ratio of BMC, fat content, and lean body content), and BMD in black-spotted pond frogs are provided in Table 1. The values of BKA (A^2^ = 28.850), BMC mass (A^2^ = 1.269), and BMC ratio (A^2^ = 0.942) had non-normal distributions (*p* < 0.05), while the values of fat content mass (A^2^ = 0.544), fat content ratio (A^2^ = 0.301), lean body mass (A^2^ = 0.328), lean body content ratio (A^2^ = 0.246), and BMD (A^2^ = 0.499) had normal distributions (*p* > 0.05). 

### 3.3. Reference Interval of Blood Serum Components

The RIs of ten serum components (glucose, AST, ALT, BUN, creatinine, TP, albumin, TGB, calcium, and phosphorus) in black-spotted pond frogs are provided in Table 2. The values of glucose (A^2^ = 2.888), AST (A^2^ = 16.220), ALT (A^2^ = 14.250), BUN (A^2^ = 15.890), creatinine (A^2^ = 13.360), calcium (A^2^ = 6.494), and phosphorus (A^2^ = 20.080) were non-normally distributed (*p* < 0.05), while the values of TP (A^2^ = 0.229), albumin (A^2^ = 0.479), and TGB (A^2^ = 0.289) were normally distributed (*p* > 0.05).

## 4. Discussion

We established the reference intervals of immunity, serum components, body composition, and BMD in 151 black-spotted pond frogs (Table S1). The RI of veterinary clinical biochemistry in some amphibians (*Xenopus laevis, Lithobates catesbeianus, Litoria caerulea, Litoria infrafrenata,* and *Cryptobranchus alleganiensis*) have been established [26,42,43,44,45]. The RI may vary depending on the life history, method and timing of blood extraction, and on the individual environmental conditions [46,47]. It is necessary to establish a more detailed RI to prepare for the variations in these conditions. In our study, we only took the samples from one species in the breeding season (May to July). RIs reflecting both male and female traits in all seasons have advantages that can be implemented extensively, but RIs established under certain environmental conditions are accurately diagnosed. The measurement of health in male frogs during the breeding season is important because the energy storage during this period affects the fertilization and hatching of eggs [36,41,48].

Studies on the RI of body composition and BMD in amphibians have rarely been carried out. Most studies use X-ray photography technology to diagnose abnormalities in individuals [49]. However, the radiography technique enables us to infer the amphibians’ health condition through an analysis of the body composition of the animals, in addition to identifying their individual status using X-ray images [50,51]. BMD can represent the nutritional, mineral, and/or food storage conditions [48,52] and body composition (such as fat and lean content) can indicate the energy storage and/or metabolic status [36,41]. Previously, we used BMD and body composition to determine the food intake status, food resource availability, and nutritional conditions of anuran species [18,27]. These compositions have interspecific variations [18], and they possess a sufficient range to detect responses to external environmental factors, such as predation pressure [27]. This radiographic technique makes it possible to diagnose an individual using accurately measured values of body composition and BMD. To diagnose these measured values on an individual basis, we require RI data.

**Figure 3:**
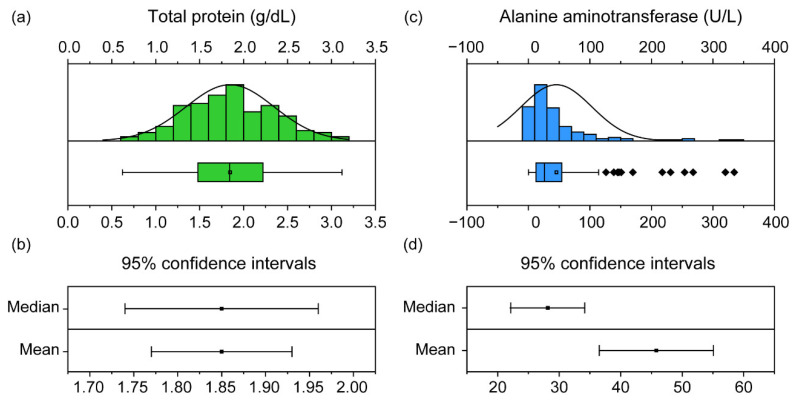
The distribution and confidence intervals (CIs) of the total protein (TP) and alanine aminotransferase (ALT) in 151 black-spotted pond frogs: (**a**) normality distribution graph and box plot of the frog TP. Box plots show the mean (central square point), median (central band), 25th and 75th percentiles (bottom and top of boxes), range within the 1.5 interquartile range (IQR) and outliers (rhombus points); (**b**) 95% confidence intervals of median and mean of TP; (**c**) non-normal distribution graph and box plot of ALT in frogs; (**d**) 95% confidence intervals for ALT median and mean.

It is important to assess the normality distribution of samples while setting RIs. Although the number of samples was sufficient in our study, each component varied in terms of its normality distribution. Commonly, in veterinary clinical biochemistry, an RI with a normal distribution is set using the 95% CI [6]. CIs can be established from both mean and median values. For components with normality (Figure 3a), the range in the CI of the mean and the CI of the median were similar (Figure 3b). Conversely, a sample without normality (Figure 3c) can vary between these two ranges (Figure 3d); therefore, a percentage value that is not affected by outliers is used instead of the CI value [29]. For this reason, the normality test is essential when establishing a reference range, and the types of reference ranges that can be used vary depending on the normality distribution.

For amphibians, the American Veterinary Medical Association (AVMA) recommends using anesthesia dissolved in water, such as MS-222 (tricaine methanesulfonate), instead of freezing anesthesia if their size is very small (<4 g) or if there is no scientific feasibility [53]. However, we determined that this method was not suitable for our study, as we measured the muscle ratio and mass, and MS-222 has been observed to affect muscle physiology [33]. Additionally, an expert group in the field of amphibians and reptiles has a positive opinion of freezing anesthesia in ectotherm animals [34]. Amphibians and reptiles from temperate regions are acclimatized to freezing conditions over their life history, depending on the season [34]. Freezing anesthesia can greatly reduce the neural activity of amphibians, which can physically and chemically anesthetize animals without signs of pain [54,55]. Hence, we adopted the method of freezing anesthesia to anesthetize frogs in our study. We believe that a specific anesthesia method, proposed for each specific animal taxon, is necessary to improve the existing anesthesia methodology, which is predominantly focused on mammals.

The components for veterinary clinical examination have tissue or area specificity. Body composition, such as BMD, muscle ratio, and fat ratio, display differences depending on the area to be measured [56,57]. In animals, biochemical enzymes have a different origin and concentration. ALT, a liver-specific indicator, is derived from the kidneys and liver, and has the highest concentration in these sites [58]. Creatinine filtered by the glomerulus differs between the urine and the blood [38]. Thus, the RI also needs to be established via the urine, blood or tissue concentrations of each enzyme. Additionally, while performing comparative analyses using these clinical components, the tissues, sites or areas must be unified.

## 5. Conclusions

Veterinary clinical examinations can be used to monitor environmental changes in the habitat and to analyze adaptation or response mechanisms in species by determining the physiological conditions of the animal. However, there are a diverse array of biological and environmental factors in habitats that can affect their physiological conditions. These factors can interact unpredictably with one another on complex spatiotemporal scales. Combined measurements of immunity, serum components, body compositions, and BMD can reduce the unpredictability caused by a wide range of environmental variables in the habitat through various time scales with a variety of potential interpretations. Usually, immune responses have a short time scale. For example, under physiological stress, the bactericidal activity in plasma responds relatively quickly (within 15 to 24 h) [59,60], and the white blood cell levels respond within one to two hours under acute stress [61,62]. These immunoassays are applied in ecoimmunology or macroimmunology to help us understand the process of disease epidemiology and the adaptive response of immune function to the environment and on a specific spatial scale [63]. Depending on the stress intensity and test components, serum components can change within 3 to 30 days and are used to identify the specific mechanisms of physiological responses to environmental changes [64,65]. Body composition and BMD change over a wider time scale range. Fat content can change within one week to two months, and BMD can change with age, over approximately one year [66,67]. These components help us to understand the ecological interactions in a habitat, including food resource availability and predation pressure [18,27], as well as energy regulation in life history processes, including growth and reproduction [68,69]. This combined examination allows us to understand the complex physiological conditions of animals. This research aligns with the goals of conservation physiology and can be used to identify the condition of species in response to environmental changes in their habitats, as well as to monitor the ecosystem.

## Figures and Tables

**Table 1:** The mean ± standard deviation (SD), median, first (25%) to third (75%) quantile range, 95% confidence interval (CI) of mean and median, and 95% percentile (2.5–97.5% range) of immunity, body composition, and bone mineral density (BMD) in 151 black-spotted pond frogs. Bacterial killing ability (BKA) was analyzed to represent the immunity. Body composition was composed of bone mineral contents (BMC), fat contents (fat), and lean body contents (lean). We calculated each body composition ratio (%) by dividing the total body weight by each body composition mass. The asterisk (*) represents the components that are not normally distributed.

Contents	Mean ± SD	Median	25%–75%	95% CI of Mean	95% CI of Median	95% Percentile
BKA (%) *	96.04 ± 6.54	97.99	96.69–98.67	94.99–97.09	97.74–98.22	69.15–99.52
BMC (g) *	0.77 ± 0.26	0.73	0.57–0.93	0.73–0.81	0.69–0.78	0.37–1.43
BMC (%) *	2.62 ± 0.57	2.55	2.22–3.01	2.53–2.71	2.45–2.68	1.73–3.98
Fat (g)	5.82 ± 1.75	5.64	4.48–7.07	5.54–6.10	5.36–6.14	2.81–9.52
Fat (%)	20.05 ± 4.15	20.28	17.12–22.94	19.39–20.72	19.36–21.22	12.09–28.46
Lean (g)	22.58 ± 5.40	22.27	18.95–25.73	21.71–23.44	21.06–23.46	12.60–34.19
Lean (%)	77.33 ± 4.13	77.18	74.65–80.25	76.66–77.99	76.23–78.07	69.00–85.18
BMD (g/cm^2^)	0.09 ± 0.01	0.09	0.08–0.10	0.08–0.09	0.08–0.09	0.07–0.12

**Table 2:** The mean ± standard deviation (SD), median, first (25%) to third (75%) quantile range, 95% confidence interval (CI) of the mean and median, and 95% percentile (2.5%–97.5% range) of ten serum components from 151 black-spotted pond frogs: glucose (GLU), aspartate aminotransferase (AST), alanine aminotransferase (ALT), blood urea nitrogen (BUN), creatinine (CRE), total protein (TP), albumin (ALB), total globulin (TGB), calcium (Ca), and phosphorus (P). The asterisk (*) represents the components that are not normally distributed.

Contents	Mean ± SD	Median	25%–75%	95% CI of Mean	95% CI of Median	95% Percentile
GLU (mg/dL) *	19.27 ± 9.96	17.60	13.14–23.02	17.67–20.87	16.3–19.06	4.86–44.66
AST (U/L) *	305.6 ± 354.7	190.1	122.2–356.6	248.6–362.7	173.3–222.7	64.38–1301
ALT (U/L) *	45.80 ± 57.73	27.08	12.32–55.74	36.51–55.08	22.14–34.16	0.80–256.7
BUN (mg/dL) *	4.16 ± 4.06	2.84	2.18–4.66	3.51–4.82	2.66–3.36	1.14–13.55
CRE (mg/dL) *	0.10 ± 0.09	0.08	0.04–0.12	0.09–0.12	0.08–0.10	0.02–0.29
TP (g/dL)	1.85 ± 0.50	1.84	1.48–2.22	1.77–1.93	1.74–1.96	0.94–2.94
ALB (g/dL)	0.52 ± 0.14	0.50	0.40–0.60	0.48–0.53	0.48–0.52	0.26–0.77
TGB (g/dL)	1.35 ± 0.39	1.32	1.04–1.62	1.28–1.41	1.24–1.42	0.64–2.20
Ca (mg/dL) *	5.66 ± 1.57	5.64	4.88–6.02	5.41–5.92	5.34–5.74	3.01–10.98
P (mg/dL) *	6.75 ± 2.98	5.90	5.36–6.66	6.27–7.23	5.82–6.20	4.10–17.52

## Data Availability

The data used for the reference intervals are available via contact with the corresponding author.

## Supplementary Materials

The following are available online at https://www.mdpi.com/article/10.3390/ani11051407/s1, Table S1: Reference intervals of immunity, serum components, body composition, and BMD in 151 black-spotted pond frogs.
